# Comparing variability in diagnosis of upper respiratory tract infections in patients using syndromic, next generation sequencing, and PCR-based methods

**DOI:** 10.1371/journal.pgph.0000811

**Published:** 2022-07-20

**Authors:** Andrew W. Bartlow, Zachary R. Stromberg, Cheryl D. Gleasner, Bin Hu, Karen W. Davenport, Shailja Jakhar, Po-E Li, Molly Vosburg, Madhavi Garimella, Patrick S. G. Chain, Tracy H. Erkkila, Jeanne M. Fair, Harshini Mukundan

**Affiliations:** 1 Bioscience Division, Los Alamos National Laboratory, Los Alamos, New Mexico, United States of America; 2 Physical Chemistry and Applied Spectroscopy, Chemistry Division, Los Alamos National Laboratory, Los Alamos, New Mexico, United States of America; 3 Medical Associates of Northern New Mexico, Los Alamos, New Mexico, United States of America; Universidad Autonoma de Baja California, MEXICO

## Abstract

Early and accurate diagnosis of respiratory pathogens and associated outbreaks can allow for the control of spread, epidemiological modeling, targeted treatment, and decision making–as is evident with the current COVID-19 pandemic. Many respiratory infections share common symptoms, making them difficult to diagnose using only syndromic presentation. Yet, with delays in getting reference laboratory tests and limited availability and poor sensitivity of point-of-care tests, syndromic diagnosis is the most-relied upon method in clinical practice today. Here, we examine the variability in diagnostic identification of respiratory infections during the annual infection cycle in northern New Mexico, by comparing syndromic diagnostics with polymerase chain reaction (PCR) and sequencing-based methods, with the goal of assessing gaps in our current ability to identify respiratory pathogens. Of 97 individuals that presented with symptoms of respiratory infection, only 23 were positive for at least one RNA virus, as confirmed by sequencing. Whereas influenza virus (n = 7) was expected during this infection cycle, we also observed coronavirus (n = 7), respiratory syncytial virus (n = 8), parainfluenza virus (n = 4), and human metapneumovirus (n = 1) in individuals with respiratory infection symptoms. Four patients were coinfected with two viruses. In 21 individuals that tested positive using PCR, RNA sequencing completely matched in only 12 (57%) of these individuals. Few individuals (37.1%) were diagnosed to have an upper respiratory tract infection or viral syndrome by syndromic diagnostics, and the type of virus could only be distinguished in one patient. Thus, current syndromic diagnostic approaches fail to accurately identify respiratory pathogens associated with infection and are not suited to capture emerging threats in an accurate fashion. We conclude there is a critical and urgent need for layered agnostic diagnostics to track known and unknown pathogens at the point of care to control future outbreaks.

## Introduction

Emerging and re-emerging infectious diseases are a threat to global health security [1] and are increasing in both frequency, scale, and severity [2–4]. Respiratory pathogens, owing to the ease of aerosol-based transmissibility, have always been associated with the most pandemic potential [5]. Outbreaks such as the recent coronavirus pandemic, the 2002 SARS (SARS-CoV) epidemic, the Middle East respiratory syndrome (MERS-CoV) epidemic in 2012, and the 2009 H5N1 influenza pandemic, demonstrate that novel and emerging respiratory viruses can cause relatively high morbidity and mortality [6]. Respiratory viruses, whether influenza or others, can spread rapidly because of increased travel and global connectivity [7, 8]. Preventing pandemic spread of an emerging infectious agent is critical for preserving global health, the global economy, and our way of life.

Viruses are the most common cause of respiratory infections, although bacterial and fungal pathogens can cause them as well [9]. Each year, in the United States alone, viral respiratory infections cause more than 400,000 hospitalizations in children less than 18 years old [10]. A subset of these upper respiratory tract infections is characterized as influenza-like illnesses (ILI), which is defined as cases of possible influenza, or other illnesses resulting in a set of symptoms that are indistinguishable from those attributed to influenza viruses. Examples of these include common cold viruses, such as rhinovirus, adenovirus, human respiratory syncytial virus (RSV), parainfluenza virus (PIV), and human metapneumovirus (hMPV) [11]. Of these, rhinoviruses are most commonly associated with the common cold [12], and have been attributed to 1/3 of the cases of respiratory infections annually in the United States [13]. In addition to these, adenoviruses and bocaviruses are also associated with respiratory infection in humans, although their prevalence in the adult population is not well known. The prevalence of these viral infections in children, where they are more prevalent, is well documented [14, 15]. It should be noted that commonly circulating human coronaviruses (CoV), a novel strain of which is responsible for the global COVID-19 pandemic today, are routinely associated with upper respiratory infections in humans. In addition to viral pathogens, several bacterial species are also associated with respiratory infections [16–19].

Common symptoms attributed to upper respiratory tract infections associated in all the above instances include cough, sore throat, runny nose, fever, chills, malaise, dry cough, loss of appetite, body aches, and nausea; combinations of which can manifest depending on various pathogen-specific, environment-specific, and host-specific factors. Thus, *syndromic diagnosis*–defined as a physician’s diagnosis based on a set of signs and symptoms of a disease (syndrome) presented by the patient–does not allow for discrimination between the various etiological agents responsible for respiratory infections [20]. Further, individuals that are asymptomatic or pre-symptomatic cannot be identified via syndromic diagnosis but may be able to transmit pathogens. Indeed, the current COVID-19 pandemic has demonstrated that reliance on syndromic diagnostics alone can result in delayed response to emerging outbreaks. Yet, syndromic diagnosis is the most used strategy for identification of infectious diseases at clinics around the world, including the United States. This is because the careful identification of an infection by a trained physician at the point of need is rapid, inexpensive, and easy to implement. Further, it is the only available strategy in the absence of a broad suite of targeted point-of-care (POC) diagnostics, which exist but only for select pathogens such as influenza A and B viruses.

As of today, a physician often has no choice but to rely on syndromic diagnosis for identification and treatment of a respiratory infection. Thus, the lack of reliable diagnostics is a major problem that complicates the identification and the effective treatment and control of emerging and known pathogens. The rationale for our work is to assess the value of targeted diagnostics, and the need for pathogen agnostic pan-diagnostics in order to address emerging threats. Here, we attempt to understand and measure efficacy of syndromic diagnosis as a method to identify and treat upper respiratory tract infections in a common clinical setting in the United States. Our study is focused on evaluating the gaps and methods for effective diagnostic surveillance using anonymized population-level estimation, so as to determine our preparedness to identify unanticipated and emerging threats. To do this, we systematically compared prevalence of upper respiratory tract infections in a given population using 1) syndromic diagnosis, 2) targeted molecular diagnostics (specifically, Polymerase Chain Reaction [PCR], and 3) untargeted pan-diagnostics (metagenomic sequencing). The outcomes of our study demonstrate the need for targeted, agnostic diagnostic strategies to facilitate the identification of atypical pathogens, asymptomatic carriers, and novel emerging threats. These findings are especially relevant in the event of an unexpected outbreak, as with the COVID-19 pandemic.

## Materials and methods

### Ethics statement

This study was designed in alignment with DOE and NIH mandated universal HIPAA guidelines. The protocol was reviewed and approved by the Institutional Review Board of the Los Alamos National Laboratory (LANL, LANL000211), in accordance with DOE Guidelines and policies, and the Department of Homeland Security’s Human Subjects Research Assessment Board. The enrollment of samples was approved as per a memorandum of understanding between the Medical Associates of Northern New Mexico (MANNM) and Los Alamos National Laboratory. Enrollees were appraised of study guidelines and processes, and informed written consent was obtained before sample collection. The consent form, that each patient was required to sign before being enrolled in the study, included information regarding sample collection, processing procedures, sample destruction after processing, and anonymity (removal of all personal identifiable information). We did not include minors in this study; all individuals were over 18 years of age. All individual information is anonymized, no identifying information is available to the LANL team members.

### Enrollment and sample collection

**Fig 1:**
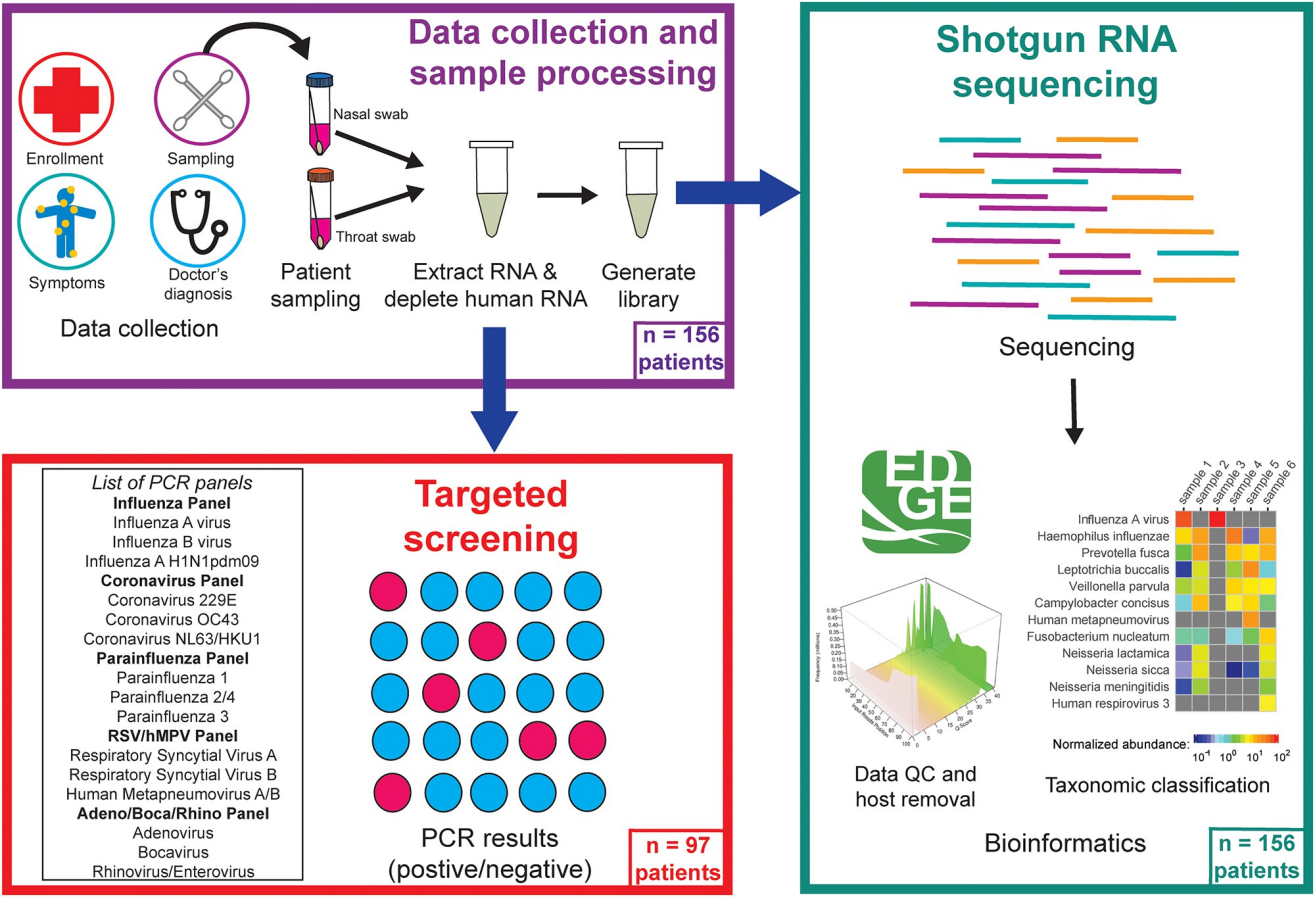
Sample collection and data processing. Individuals at the clinic were enrolled in the study and self-reported their symptoms. Nasal and throat swabs were then collected from individuals, from which RNA was extracted and human RNA was depleted. Extracted RNA was used in the PCR panels (97 individuals), while libraries were created for samples from all individuals and sequenced using an Illumina NextSeq. The shotgun RNA sequencing reads were analyzed using EDGE Bioinformatics and various taxonomic tools implemented within EDGE.

A flowchart of the data collection and sample processing, as performed at MANNM Clinic, Los Alamos, New Mexico, is shown in Fig 1. In 2018, individuals were sampled from 31 January to 10 April. In 2019, individuals were sampled from 6 February to 9 April. We selected individuals opportunistically from a typical hospital setting in northern New Mexico for two years, during the annual influenza season cycles. This patient pool is diverse in terms of both gender and age groups and is representative of a community in the state of New Mexico. Thus, the sample represents the diversity of a normal population in an American town. An intrinsic bias of our study is that only individuals that visited MANNM were sampled. However, this population included individuals that were either healthy (e.g., annual physical check-ups and other routine appointments), sick with something unrelated to respiratory pathogens (e.g., non-infectious diseases like diabetes, cardiovascular issues), and sick with symptoms of respiratory infection. For the purpose of this study, individuals that were completely healthy, or sick with conditions unrelated to respiratory infections, are both defined as controls, whereas individuals presenting with symptoms of respiratory infections are categorized as sick. Sick individuals were defined as individuals presenting with one of the following common symptoms of upper respiratory tract infections: cough, sore throat, runny nose, and fever, all of which may lead to ILI presentation. Several studies have demonstrated that atypical presentations of ILI (e.g., no fever), that do not conform to the WHO/CDC definition, are common in people with influenza and other respiratory illnesses [21]. Documentation of fever, or measured fever at the time of enrollment, was considered too restrictive for this study, especially for capturing those patients who may have taken antipyretics to reduce fever, patients who do not present with fever, as often seen in adults. Further, we wanted to capture atypical pathogens and emerging threats that may not confirm to the conventional definition of ILI.

Individuals volunteered for the study after being approached by staff in waiting rooms or contacted the LANL team after seeing flyers around the hospital and/or notifications on the LANL website and daily bulletins. Individuals visiting the clinic for routine appointments were enrolled in the study prior to their scheduled appointment with the physician. Consenting individuals were first asked to complete a self-assessment questionnaire, in order to obtain data to the analysis of diagnostics outcomes, but that did not compromise individual identity or collect health criteria unrelated to respiratory infections. Individuals were asked if they presented with any of the following broad range of symptoms: cough, sore throat, fever, runny nose, ear ache, body aches, weakness and fatigue, headache, congestion, and asthma/wheezing. The self-assessment questionnaire also allowed for the determination of sex, age-group, influenza vaccination status (i.e., did they receive an influenza vaccine for the given season), and the reason for their visit to the clinic. In the 2018 study phase, two nose and two throat swabs were taken from sick and control individuals. However, in 2019, nose and throat swabs were collected only from sick individuals.

Two nose and throat swabs were collected from every individual using optimized protocols as described here. In order to reduce contamination and maintain sterile conditions, the collection area, ice packs, and the transportable cooler were cleaned with 10% bleach (2 min) followed by 70% ethanol (10 min, evaporation). We used sterile HydraFlock swabs (25-3406-H; Puritan Medical Products) for both the nasal and throat samples. All samples were placed in sterile 15 mL Corning Falcon Conical Centrifuge Tubes (ThermoFisher Scientific, Cat. #14-959-53A) in TRIzol LS (Life Technologies, ThermoFisher Scientific, Cat. #10296010). The focus on RNA extraction eliminated the thorough understanding of DNA viruses and several bacterial pathogens, which is a second intrinsic bias of our study design.

In the first year of the study, we attempted to understand the relative efficacy of throat vs. nasal swabs for diagnosis of respiratory infections. Therefore, in 2018, two nose swabs and two throat swabs from each individual were collected and each placed in 2 mL of TRIzol. Preliminary analysis of outcomes showed that the RNA yield and combined efficacy of throat and nasal swabs was more reliable and sensitive than either sample alone. Therefore, in 2019, four swabs (two nose and two throat) were still collected per patient, but all of them were placed in one single 15 mL tube with 2 mL of TRIzol. The tubes were vortexed twice for 10s each in order to ensure thorough suspension of the sample in TRIzol, with a 3 min interim incubation period. The tubes were then wiped down with 10% bleach followed by 70% ethanol, labeled, and placed in a cooler with ice packs. Samples were transported to the laboratory and stored at -80°C within 4 h of being collected. Samples were removed only for RNA extractions, and extracted RNA was used for both shotgun RNA sequencing and PCR assays as described below.

Subsequent to sampling, individuals were seen by the physician at the clinic, and a clinical diagnosis was made where applicable. The research team worked with the administrative assistants at MANNM in order to collect diagnostic information pertinent to the study. Specifically, a positive diagnosis of respiratory infection by the physician (syndromic diagnosis) and outcomes of POC diagnostic assays performed, if any, were recorded. These diagnoses were obtained from patient records following the appointment. In most individuals, specific diagnosis of the pathogen was not made at the clinic, although the individual was confirmed to have respiratory infection for which they were either treated or advised care as appropriate. This means that a pathogen was not explicitly identified as the causative agent. For individuals that were confirmed to have a respiratory infection or viral syndrome, this was recorded as "yes" for respiratory infection diagnosis. The POC diagnostics information included both lateral flow strip tests performed on site, and PCR-based evaluations at a regional laboratory.

### RNA extraction

Swabs were stored at -80°C in TRIzol until RNA was extracted using the Direct-zol MiniPrep Plus Kit (Zymo Research, Cat. #R2071, USA), following manufacturer instructions. Initially, as described earlier, nasal and throat swabs were extracted and analyzed separately (n = 22). Based on the RNA yield, and the need for consistent samples for both genomic and PCR-based assessment, all subsequent samples were pooled together. For the nasal and throat swabs that were extracted and analyzed separately, the PCR and RNA-seq results were combined for each patient. The concentration of total RNA was measured using the Qubit RNA HS Assay Kit (ThermoFisher Scientific, Cat. #Q32855, USA), and quality of RNA was assessed using the Bioanalyzer High Sensitivity DNA Kit (Agilent, Cat. #5067–4626, USA). The extracted RNA was used for both PCR and shotgun RNA sequencing.

### PCR for respiratory viruses

Based on prevalence information in Northern New Mexico, we targeted the following seven respiratory viruses: influenza A, influenza B, coronavirus, hMPV, PIV, RSV, and rhinovirus/enterovirus. Pooled nucleic acid (5 μl per reaction) extracted from nasal and throat swabs was tested using the RealAccurate Quadruplex Respiratory PCR one-step reverse transcriptase qPCR kits (PathoFinder, Maastricht, Netherlands) as per the manufacturer’s instructions (Fig 1). Assays for the different viruses were performed using the following panels: influenza panel (influenza A and influenza B), coronavirus panel (coronavirus 229E, coronavirus OC43, and coronavirus NL63/HKU1), parainfluenza panel (PIV 1 to 4), RSV A and B and hMPV panel, and an adeno/boca/rhino panel (adenovirus, bocavirus, rhinovirus/enterovirus). Adenovirus and bocavirus are both DNA viruses and not expected to be detected because of use of RNA extraction in this study. The Adeno/Boca/Rhino panel was performed because rhinovirus/enterovirus are RNA viruses, and diagnostics are available only as part of this panel. These reactions were performed on an ABI 7500 Fast Dx instrument. The following amplification conditions were used: 50°C for 10 min, 95°C for 1 min, and 40 cycles of 95°C for 10 s and 60°C for 1 min. An internal control provided by the manufacturer was used in each reaction, and positive and negative controls provided by the manufacturer were used in each run. The cycle threshold (Ct) cutoff for a positive result for each panel was 40. A Ct value lower than 25 was considered a strong positive sample, whereas a Ct value between 35 and 40 was considered a weak positive sample.

### Library preparation and shotgun RNA sequencing

Ribosomal RNA was depleted using Ribo-Zero H/M/R Assay (Illumina, Cat. #RZH1046, USA). The concentration of depleted RNA was obtained using the Qubit RNA HS Assay Kit (ThermoFisher Scientific, Cat. #Q32855, USA) and quality was obtained using the Bioanalyzer High Sensitivity DNA Kit (Agilent, Cat. #5067–4626, USA). RNA was converted to cDNA and adapters and indexes were added to the ends of the fragments to generate Illumina libraries using the KAPA Hyper RNA Prep Kit (KAPA Biosystems, Cat. #KK8541 and KA4000, South Africa). Illumina libraries were eluted in DNA Elution Buffer (Zymo Research, Cat. #D3004-4-10, USA). The concentration of the libraries was obtained using the Qubit dsDNA HS Assay (ThermoFisher Scientific, Cat. #Q32854, USA). The average size of the library was determined by the Agilent High Sensitivity DNA Kit (Agilent, Cat. #5067–4626, USA). Accurate library quantification was performed using the Library Quantification Kit–Illumina/Universal Kit (KAPA Biosystems, Cat. #KK4824, South Africa). Libraries were normalized to the same concentration based on the qPCR results. Specifically, the libraries were normalized to 2 nM prior to denaturing and diluting to a final loading concentration of 1.5 pM. Each library was sequenced on approximately three percent (24 million reads) of a NextSeq High Output flow cell to generate paired-end 151 bp reads using the NextSeq 500/550 High Output Kit v2.5 Kit (300 cycles) (Illumina, Cat. #20024908, USA).

### Bioinformatic analysis

We used the Los Alamos National Laboratory’s EDGE Bioinformatics platform [22] to process all RNA shotgun sequencing results. Illumina FASTQ files were imported to EDGE. All samples were run as a batch submission; and therefore, treated the same way. For pre-processing, quality trim and filter were applied using a trim quality level of 20 and a minimum read length of 50 bp. Host nucleic acid removal was performed using the human GRCh38 reference genome with a 90% similarity cutoff to ensure removal human RNA.

Shotgun sequencing is agnostic to targets (unlike PCR), but enough sequencing depth is required to accurately identify pathogens, especially if they are present in low abundance. To identify sequencing reads with similarity to known pathogens, we used read-based taxonomic classification using a combination of two taxonomic identification tools developed at LANL. We used Genomic Origin Through Taxonomic CHAllenge (GOTTCHA2) [23] and the novel Pan-Genomics for Infectious Agents (PanGIA) tool [24], which differ in their sensitivity and specificity to identify pathogens. GOTTCHA2 uses the minimap2 alignment tool, while PanGIA uses BWA-MEM for alignments. Because they are read-based taxonomic tools, there is no assembly involved. Both tools were implemented in EDGE and are based on genomic alignment to bacterial and viral species. Whereas PanGIA has enhanced sensitivity over GOTTCHA2, the latter is more specific as it exclusively utilizes unique genomic signatures for profiling, and therefore has fewer false positives [24]. We first used PanGIA to scan for the seven common NM viruses. If a virus was detected using PanGIA, the outcome was confirmed using the more specific taxonomic tool, GOTTCHA2. If GOTTCHA2 did not identify a virus, then we examined the linear genomic coverage in order to determine the distribution of reads that mapped to the reference genomes and determine presence based on this comparative analysis rather than any one tool alone. We believe that this iterative approach offers greater sensitivity and specificity of outcomes than either alone, a factor which is critical when examining emerging and reemerging pathogens identified in widely divergent human populations. This approach also allows the identification of normal human microbiota and can discriminate pathogens from closely related species and strains [24]. PanGIA was also used to identify any bacterial species in the samples.

When discrepancies were identified between PCR and shotgun RNA sequencing, we manually analyzed the sequencing results in order to verify outcomes. This allowed for identification of true pathogens and minimized false positives significantly. Manual verification involved mapping the putative pathogen-derived reads against all reference genomes in NCBI (GenBank entry: K02121.1 and FJ445111.1 and refseq: all coronavirus genomes) using Minimap2, which is the most common method for mapping Illumina pair-end reads.

We used PCR diagnostics as confirmatory measurements, and NGS as an agnostic catch-all pan-diagnostic to explore for pathogens that may not be targeted by PCR panels. The sensitivity and reliability of PCR assays for respiratory pathogen detection has been well established, and it has been argued that they should form the front line surveillance methods for early identification of emerging respiratory infections [25]. Sequencing has been proposed as a pan-diagnostic strategy, and has been noted to be highly effective in capturing unanticipated pathogens, including coronaviruses, in clinical samples [9].

### Comparison of syndromic diagnosis with PCR and RNA sequencing

We determined the true positives, true negatives, false positives, and false negative outcomes from the individual PCR and sequencing data. In order to assess the variability in the three methods, we first report the percent of individuals with upper respiratory tract infection symptoms that were not diagnosed to have an upper respiratory infection or a viral syndrome by syndromic diagnostics at the physician’s office. We then describe those cases where the syndromic diagnosis did not correlate with the findings by PCR and shotgun RNA sequencing. These two metrics are described as an indication of the need for more targeted methods to capture the variety of respiratory pathogens in human populations.

## Results

### Enrollment study information

We enrolled and sampled 132 individuals in 2018, and 24 in 2019, for a total of 156 for this study (S1 Data). Healthy volunteers, or anyone who had medical needs beyond upper respiratory infections (e.g., diabetes, high blood pressure, and others) were categorized as controls at the time of enrollment. Individuals were categorized as sick based entirely on self-identification of symptoms associated with respiratory infections at the time of enrollment. Based on this initial categorization of all enrollees, 97 individuals (2018 and 2019) were ‘sick’; and 59 were ‘controls’ or ‘not sick’ (Table 1). This early categorization was performed exclusively for the purpose of binning samples for analysis and further evaluations and did not influence subsequent diagnostics. For instance, it is noted that the control category may include potential asymptomatic carriers and individuals with pre-symptomatic presentation of the disease; and the sick category can include individuals with seasonal allergies that often manifest similar to respiratory infections.

**Table 1:** Demographic and clinical characteristics of the 156 individuals sampled, split into sick and control (non-sick) groups.

	Sick group based on symptoms (n = 97)	Control group based on lack of symptoms (n = 59)
Age (no., %)		
18–27	8 (8.2%)	4 (6.8%)
28–37	14 (14.4%)	11 (18.6%)
38–47	17 (17.5%)	8 (13.6%)
48–57	15 (15.5%)	13 (22.0%)
58–67	18 (18.6%)	13 (22.0%)
> 67	25 (25.8%)	10 (16.9%)
Sex (no., %)		
Female	55 (56.7%)	28 (47.5%)
Male	41 (42.3%)	30 (50.8%)
Did not answer	1 (1.03%)	1 (1.7%)
Received flu shot (no., %)	69 (71.1%)	47 (79.7%)
Upper respiratory infection symptoms (no., %)^a^	97 (100%)	0
Cough	68 (70.1%)	0
Sore throat	52 (53.6%)	0
Runny nose	67 (69.1%)	0
Fever	14 (14.4%)	0

^*a*^Percentages do not add up to 100 for the individual symptoms because patients often had more than one symptom. Upper respiratory tract infections are defined as having at least one of the following symptoms: cough, sore throat, runny nose, and fever.

Of the 156 individuals enrolled in this study, 97 (62.2%) presented with symptoms of respiratory infection (as defined earlier for the purpose of this study), while 59 (37.8%) did not present with any symptoms of respiratory infection (Table 1). The demographic characteristics (age groups and gender) are shown in Table 1 for both 2018 and 2019. Also shown are the numbers and percentages of individuals that received the annual influenza vaccination (each year) as well as numbers and percentages of individuals with symptoms of upper respiratory infection.

### Comparison of nasal and throat swabs

The microbial profiles of nasal and throat samples, collected separately, were compared for 22 individuals sampled in 2018, using shotgun RNA sequencing. Seventeen of 22 (77.3%) individuals had symptoms of respiratory infection with targeted viruses being detected in 7 (31.8%) individuals only. The rest of the individuals (n = 15; 63.6%) were negative for all targeted viruses in both the nasal and throat samples.

**Fig 2:**
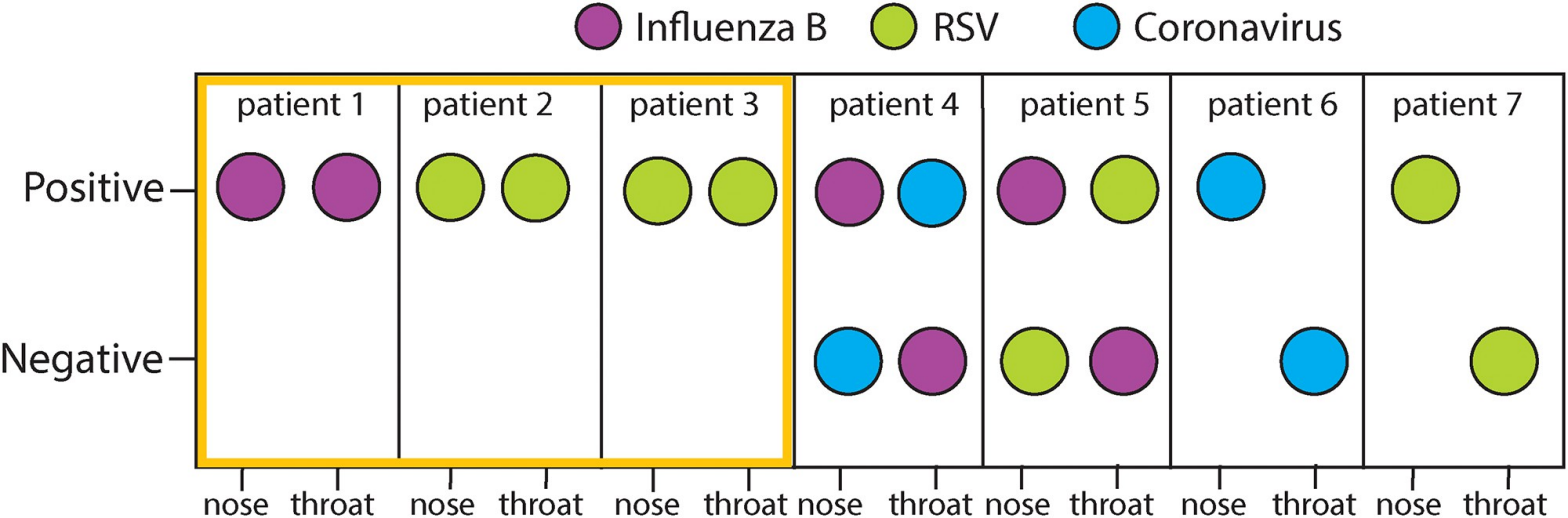
Summary of nose and throat comparison using shotgun RNA sequencing. Nose and throat samples were analyzed separately in 22 individuals. Seven out of 22 individuals were positive in at least one of the samples. The same virus was detected in both types of samples in 3 individuals (orange box). The other 5 individuals showed differences between viruses detected in the nose versus the throat.

Out of the 7 individuals in whom one or more of the 7 targeted viruses were detected, only 3 (42.8%) had consistent findings between the nasal and throat samples. Of these 3 individuals, 1 contained influenza B and 2 contained RSV (Fig 2). In the other 4 individuals tested, different results were found in nasal and throat samples. Target viruses were found in all 4 nasal samples, influenza B virus in 2 individuals, and RSV and coronavirus in the others (Fig 2). In the corresponding throat samples, both influenza B individuals yielded coronavirus and RSV, and no viral targets were found in the other 2 throat samples (Fig 2). Different samples have been used for diagnosis of respiratory pathogens and the discrepancy between outcomes has been previously noted [26].

### Results of individual diagnostic platforms evaluated

#### PCR

From a total of 156 pooled nasal and throat pooled samples, 97 were randomly selected for testing by commercial PCR assays for common respiratory viruses. By PCR, it was determined that 21 out of 97 samples were positive for one or more respiratory viruses (Table 2). Eighteen of these 21 (85.7%) individuals presented with upper respiratory symptoms. Three individuals (14.3%) did not have symptoms of respiratory infections but were positive for a virus by PCR. Of the 21 positives, 8 samples were positive for coronavirus, 4 for RSV, 3 for PIV, 4 for influenza virus (2 for influenza A and 2 for influenza B), 2 for rhinovirus/enterovirus, and 1 for hMPV (Table 2).

**Table 2:** Respiratory viruses detected by PCR and shotgun RNA sequencing. For the PCR panels, the Ct cutoff for a positive result was 40 and a value between 35 and 40 indicated a weak positive sample.

Sample	PCR Result	CT score^a^	RNA-seq Result^b^	# reads (linear coverage)^c^	Upper respiratory infection symptoms^d^
120	Influenza A	16.23	Influenza A	501,424 (100%)	Yes
156	Influenza A	25.68	Influenza A	709 (93%)	Yes
026	Influenza B	19.25	Influenza B	123,943 (100%)	Yes
119	Coronavirus	24.75	Coronavirus	74 (30%)	Yes
154	Coronavirus	17.75	Coronavirus	5,647 (99%)	Yes
128	Coronavirus	25.01	Coronavirus	14,999 (99%)	Yes
138	Coronavirus	22.42	Coronavirus	338,095 (99%)	Yes
080	Coronavirus, RSV	17.77; 28.34	Coronavirus, RSV	34,963 (100%); 6 (6%)	Yes
132	Parainfluenza	27.63	Parainfluenza	8,635 (96%)	Yes
135	Parainfluenza	22.65	Parainfluenza	727 (90%)	Yes
137	Parainfluenza	24.25	Parainfluenza	454 (80%)	Yes
133	Metapneumovirus	21.50	Metapneumovirus	582 (63%)	Yes
052	Influenza B	22.80	Influenza B, **RSV**	51,024 (100%); 199 (71%)	Yes
131	RSV	31.92	RSV, **Parainfluenza**	139 (60%); 11 (5%)	Yes
033	**Coronavirus**	35.99	Negative	0 (0%)	Yes
059	**Coronavirus**	35.47	Negative	0 (0%)	No
146	**Coronavirus**	37.11	Negative	0 (0%)	Yes
014	**RSV**	31.25	Negative	0 (0%)	Yes
054	**RSV**	25.53	Negative	0 (0%)	No
090	**Rhinovirus**	34.70	Negative	0 (0%)	No
122	**Rhinovirus**	32.62	Negative	0 (0%)	Yes

^*a*^The cycle threshold (CT score) is listed for PCR.

^*b*^Bold denotes the viruses that were detected by shotgun RNA sequencing that were not detected using PCR and *vice versa*.

^*c*^The number of reads and linear coverage is listed for the shotgun RNA sequencing using the PanGIA taxonomic tool.

^*d*^Upper respiratory tract infections are defined as having at least one of the following symptoms: cough, sore throat, runny nose, and fever.

#### RNA sequencing

From a total of 156 nasal and throat pooled samples, we detected at least one of our target viruses in 23 samples using RNA sequencing. Out of the 21 individuals that tested positive using PCR, RNA sequencing completely matched in only 12 (57%) of these individuals (Table 2). All 23 individuals that tested positive for one target virus by sequencing had presented with symptoms of respiratory infection (Table 3). Of the 23, 7 were positive for influenza virus (3 for influenza A and 4 for influenza B), 7 for coronavirus (strains HKU1, 229E, and NL63), 1 for hMPV, 4 for PIV, 8 for RSV, and 0 for rhinovirus. Four individuals were infected with 2 viruses, resulting in a total of 27 viruses detected in the 23 patients. Of the 7 individuals positive for influenza virus, 5 had received a flu vaccine in that year (4 from 2018 and 1 from 2019). This is consistent with the observation that the influenza annual epidemic was especially severe in 2018, where the predominant circulating influenza A strain was H3N2, the most commonly circulating strain since the 2009 H1N1 epidemic. H3N2 is much harder to immunize against and the vaccine efficacy for this strain was reported to be between 12–31% for 2017–2018 [27].

**Table 3:** PCR results, shotgun RNA sequencing results, and physician’s diagnosis in individuals with symptoms of respiratory infection (n = 52). Individuals not included here (n = 45) had symptoms of respiratory infection, but did not have a physician’s diagnosis or a POC test, and were negative by both PCR and/or sequencing. Two individuals did not have symptoms, or a diagnosis, of upper respiratory infection, but were positive for a virus. Five additional individuals were given a diagnosis of upper respiratory tract infection even though they did not present with upper respiratory infection symptoms focused on here.

Sample	Symptoms^a^	Physician’s diagnosis	Upper respiratory infection diagnosis (Y/N)	POC tests^b^	Viruses detected with PCR	Viruses detected with RNA seq
120	cough, sore throat, runny nose, fever	influenza A	Y	flu test = pos. (A)	influenza A	influenza A
020	cough, sore throat, runny nose, fever	flu-like symptoms, non-recurrent frontal sinusitis	Y	flu test = neg.	not tested	coronavirus
045	cough, sore throat, fever	sore throat, seasonal allergic rhinitis	Y	NA	negative	negative
049	cough, sore throat, fever	cough	N	NA	negative	negative
044	cough, sore throat, fever	flu-like symptoms, acute maxillary sinusitis	Y	flu test = neg.	not tested	negative
129	cough, sore throat, fever	influenza, bronchitis	Y	NA	not tested	negative
136	cough, sore throat, fever	acute non-recurrent pansinusitis	Y	NA	negative	negative
080	cough, sore throat, runny nose	none	N	NA	coronavirus, RSV	coronavirus, RSV
154	cough, sore throat, runny nose	none	N	NA	coronavirus	coronavirus
135	cough, sore throat, runny nose	acute rhinosinusitis	Y	NA	parainfluenza	parainfluenza
132	cough, sore throat, runny nose	cough, acute upper respiratory infection	Y	NA	parainfluenza	parainfluenza
047	cough, sore throat, runny nose	none	N	NA	not tested	RSV
084	cough, sore throat, runny nose	none	N	NA	not tested	influenza A
144	cough, sore throat, runny nose	viral pharyngitis	Y	NA	not tested	RSV
015	cough, sore throat, runny nose	none	N	NA	not tested	RSV
027	cough, sore throat, runny nose	acute recurrent maxillary sinusitis	Y	NA	not tested	RSV
128	sore throat, fever, runny nose	none	N	NA	coronavirus	coronavirus
156	cough, sore throat	throat pain, URI	Y	NA	influenza A	influenza A
052	cough, sore throat	cough	N	NA	influenza B	influenza B, RSV
119	cough, sore throat	pharyngitis	Y	NA	coronavirus	coronavirus
137	cough, sore throat	viral syndrome, sinusitis	Y	NA	parainfluenza	parainfluenza
121	cough, sore throat	acute non-recurrent pansinusitis	Y	NA	negative	negative
122	cough, sore throat	upper respiratory infection, acute pansinusitis	Y	NA	rhinovirus	negative
014	cough, sore throat	none	N	NA	RSV	negative
125	cough, sore throat	sore throat, flu-like symptoms	Y	flu test = neg.	negative	negative
127	cough, sore throat	sore throat, pharyngitis	Y	NA	negative	negative
130	cough, sore throat	flu-like symptoms, acute nasopharyngitis	Y	flu test = neg.	negative	negative
142	cough, sore throat	viral syndrome	Y	NA	negative	negative
134	cough, sore throat	abdominal pain	N	NA	not tested	negative
055	cough, sore throat	flu with pneumocystis pneumonia	Y	NA	negative	negative
149	cough, sore throat	acute otitis media, acute rhinosinusitis, flu symptoms	Y	flu test = neg.	negative	negative
064	cough, sore throat	cough, sore throat	Y	NA	not tested	negative
126	cough, sore throat	acute sinusitis, flu-like symptoms	Y	flu test = neg.	not tested	negative
131	cough, runny nose	viral syndrome	Y	NA	RSV	RSV, parainfluenza
050	cough, runny nose	non-supportive otitis media of left ear, acute max sinusitis	Y	NA	negative	negative
007	cough, runny nose	none	N	NA	not tested	RSV
008	cough, runny nose	none	N	NA	not tested	influenza B, coronavirus
141	cough, fever	respiratory infection	Y	NA	negative	negative
088	cough, fever	acute non-recurrent sinusitis, bronchitis	Y	NA	not tested	negative
091	sore throat, runny nose	acute non-recurrent max sinusitis	Y	NA	negative	negative
001	sore throat, fever	sore throat, flu-like symptoms, uvulitis	Y	flu test = neg.	not tested	negative
026	sore throat	viral pharyngitis	Y	NA	influenza B	influenza B
151	sore throat	acute sinusitis	Y	NA	negative	negative
133	cough	respiratory infection	Y	NA	metapneumovirus	metapneumovirus
146	cough	cough/URI	Y	NA	coronavirus	negative
033	cough	none	N	NA	coronavirus	negative
153	cough	cough	N	NA	negative	negative
063	cough	cough with atypical pneumonia	Y	NA	negative	negative
028	cough	none	N	NA	not tested	influenza B
061	cough	sore throat, sinusitis, bronchitis	Y	flu test = neg.	not tested	negative
152	cough	COPD exacerbation, URI	Y	NA	not tested	negative
138	runny nose	none	N	NA	coronavirus	coronavirus
054	none	none	N	NA	RSV	negative
059	none	none	N	NA	coronavirus	negative
090	none	pharyngitis	Y	NA	rhinovirus	negative
140	none	flu-like symptoms, influenza A, was given antivirals	Y	flu test = pos. (A)	negative	negative
012	none	viral upper respiratory infection, given Tamiflu	Y	NA	negative	negative
036	none	acute non-recurrent max sinusitis,cough	Y	NA	negative	negative
150	none	influenza	Y	NA	negative	negative

^*a*^Upper respiratory infection symptoms are defined as having at least one of the following symptoms: cough, sore throat, runny nose, and fever. Similar symptoms are organized together.

^*b*^Included are any point-of-care (POC) tests done during the visit to the clinic.

Four individuals were co-infected with 2 viruses. For instance, one individual tested positive for both influenza B and RSV by sequencing. This demonstrates, along with the nose and throat comparison, that two respiratory pathogens can be found in one individual, potentially exacerbating symptoms. All individuals that had been categorized as controls at the time of enrollment were negative for all the targeted respiratory viruses by shotgun RNA sequencing.

**Fig 3:**
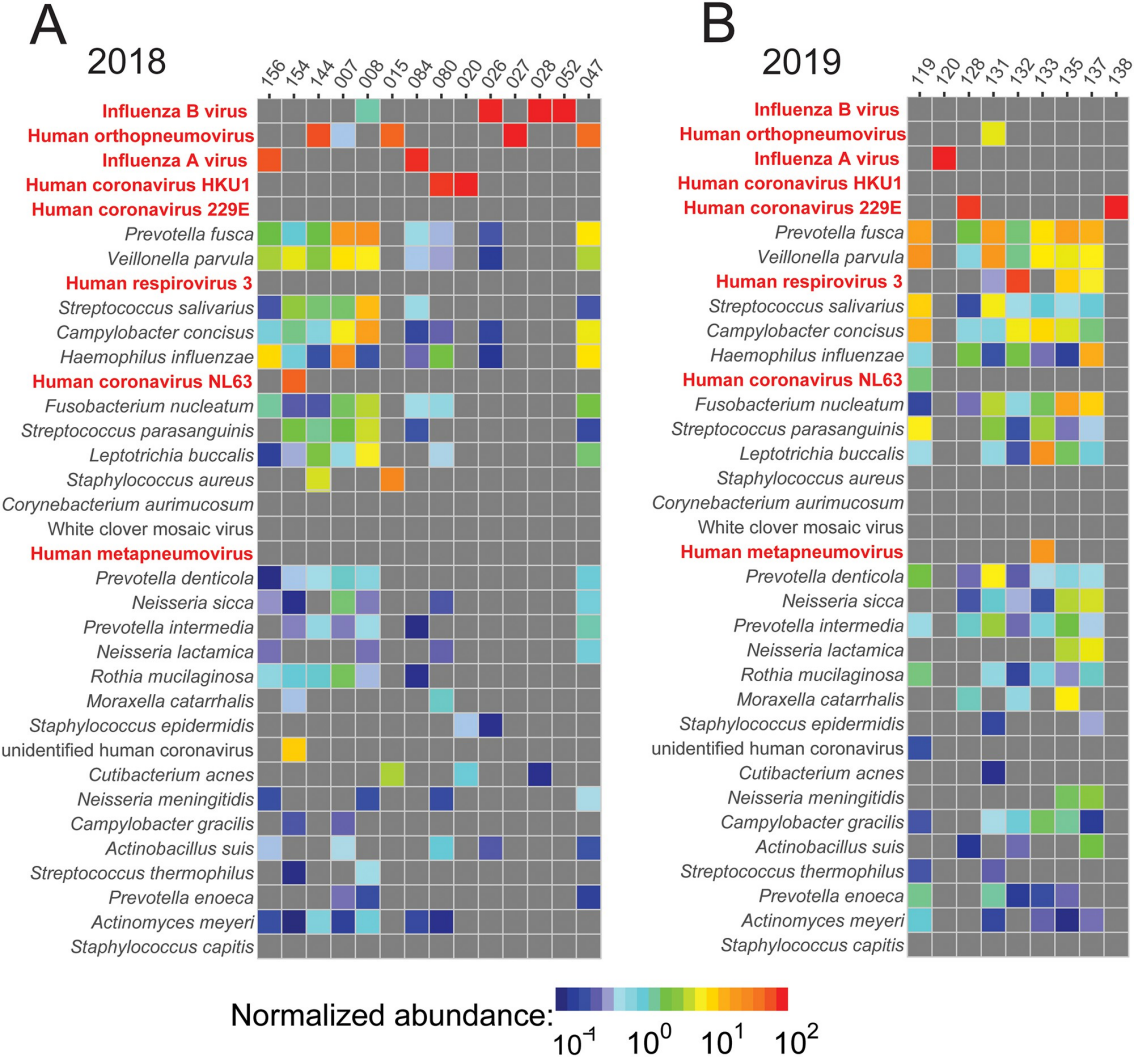
Heat maps of target viral pathogens (species in red) and other viral and bacterial species from (A) 2018 and (B) 2019 from RNA sequencing using the PanGIA taxonomic tool. Species are ordered from most abundant to least abundant. Abundance is normalized and is designated by color (red colors refer to more abundant species, while blue colors refer to least abundant species).

This target-agnostic detection capability of sequencing-based testing is a definite advantage of this platform for identification of ‘unknown’ future outbreaks and threats. We also evaluated the ability of this technique to identify bacterial pathogens, although the study design was focused on viral targets, specifically RNA viruses. RNA sequencing and the associated PanGIA taxonomic ID tool also identified several bacterial pathogens [24]. The 5 most common bacterial species detected using this method were *Prevotella fusca*, *Veillonella parvula*, *Streptococcus salivarius*, *Campylobacter concisus*, and *Haemophilus influenzae* (Fig 3). Because RNA was sequenced rather than DNA, the normalized abundance for each species of bacteria is typically lower than the abundance of each virus, which was an anticipated outcome. We only sequenced transcribed RNA, which results in lower linear coverage and depth of coverage when aligning to reference genomes. The purpose of this exercise was only to determine the feasibility of sequencing-based technologies to be adapted to various types of pathogens in the future, if required. The top 5 viral species identified by sequencing matched the strains that were being targeted by PCR, namely influenza B, human orthopneumovirus (RSV), influenza A, human coronavirus HKU1, and human coronavirus 229E.

#### Syndromic

Of the 97 individuals with symptoms of upper respiratory tract infection, 36 individuals (37.1%) were diagnosed to have an upper respiratory tract infection or a viral syndrome (e.g., acute nasopharyngitis) by syndromic diagnostics at the physician’s office (Table 3). However, the types of viruses could not be distinguished in all these cases, except for one (Table 3). In this case, the patient was diagnosed with influenza A by a rapid POC test, having presented with all 4 upper respiratory tract infection symptoms (cough, sore throat, runny nose, and fever). Five patients were given a diagnosis of upper respiratory tract infection or viral syndrome even though they did not present with the four upper respiratory tract infection symptoms we focused on (Table 3). These patients had other symptoms, such as body pain, fatigue, and headache. With the limited availability of POC tests at the physician’s clinic and the need for timely diagnostic outcomes, syndromic diagnostics are the only available option for many health care facilities. Below, we present a comparison of this assessment with confirmatory diagnostic methods such as PCR and sequencing.

### Comparison of PCR, RNA sequencing, and syndromic diagnosis

The outcomes of PCR and RNA sequencing were systematically compared with each other in order to evaluate alignment (Table 2). A 90.7% agreement (88/97, with 9 disagreements) was observed between the two methods for the 97 clinical samples compared. Of the nine disagreements, 7 were negative by shotgun RNA sequencing, but positive using PCR, as outlined in Table 2. Two samples were positive by RNA sequencing, but negative by PCR (one sample positive for PIV and one for RSV; Table 2). Thus, PCR detected one or more viruses in 21 individuals, while RNA sequencing detected one or more viruses in 14 individuals (Table 2).

**Fig 4:**
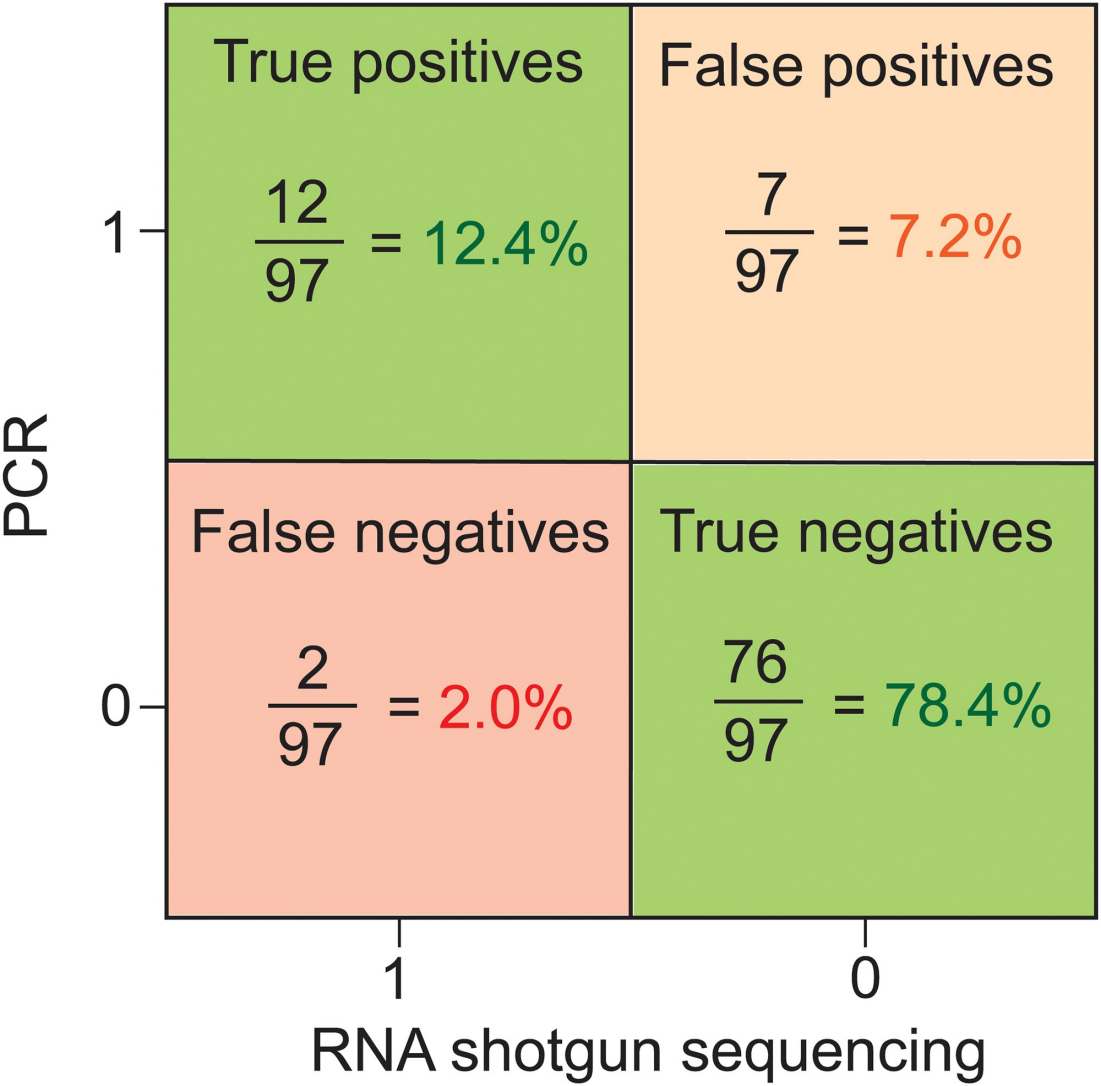
Accuracy of PCR and RNA metagenomic sequencing. RNA shotgun sequencing was used as the ‘true’ reference test. In cases with co-infections, true positive means that both diagnostic approaches found both viruses.

A systematic comparison of outcomes can be made by assuming one of the two diagnostic modalities as the "true" reference test. For instance, if the RNA sequencing were considered as the ‘true’ reference test, then a comparison of the outcomes of the PCR assay against this standard is shown in Fig 4. Twelve of 97 patients were positive for a viral infection using both PCR and sequencing, indicating a true positive rate of 12.4%. Out of the 12 positives, all 12 individuals had symptoms of upper respiratory tract infection (Table 2). Seventy-six of 97 individuals were negative by both methods, indicating a true negative rate of 78.4%. However, 7 individuals were only positive by PCR, indicating a false positivity rate of 7.2%, and two individuals were diagnosed only by sequencing, indicating a false negativity rate of 2.0% (Fig 4). If we used PCR as the ’true’ reference test, the false negativity rate was 7.2%. Because both methods are highly reliable in the way we are conducting them, we believe that a positive from either method is a true positive, and using this metric is a more accurate representation of the false negativity rate.

Broken down by viruses, those showing the biggest discrepancies between PCR and RNA sequencing were coronavirus, rhinovirus, and RSV. Three samples (033, 059, 146) were positive for coronavirus using PCR, and negative by shotgun RNA sequencing. To further evaluate these results, all the species level coronavirus reference sequences were collected from refseq v90, and the reads from the three samples were mapped against them using Minimap2. While some reads did align to some coronavirus references, all of the mapped regions were of low complexity (e.g., almost all of them were poly-As). Furthermore, not surprisingly, the entire length of these read was not aligned; thus, we conclude that these viruses are not found using RNA sequencing. We hypothesize that without any enrichment, the viral titers in these three samples were possibly too low for a reliable outcome by shotgun sequencing. This is consistent with the qPCR CT scores (Table 2) for these samples, which were >35.4. For these PCR panels, the Ct cutoff for a positive result was 40 and a value between 35 and 40 indicated a weak positive sample. It is unclear if these results point toward low viral burden in these samples and false negative results via sequencing, or potential off-target amplification resulting in a PCR false positive.

Discrepancies were found in six other samples (014, 052, 054, 090, 122, and 131) between PCR and NGS sequencing paired with EDGE Bioinformatics analysis. Specifically, PCR detected RSV in samples 014 and 054. However, no sequencing reads from samples 014 and 054 could be aligned to RSV reference genomes in a manual bioinformatics analysis (using Minimap2), or using the EDGE platform, which suggests that there were no RSV sequences in the dataset or that the virus was not sequenced. Similarly, PCR detected rhinovirus in samples 090 and 122. However, the only two reads from Sample 090 that could be aligned to rhinovirus reference genome were singletons (only one read of a read pair set can be aligned to the reference), which are considered alignment issues. Sample 122, on the other hand, had 94 paired reads that were able to be manually aligned to rhinovirus. However, all these reads aligned poorly to one or both of the GenBank references (GenBank entry: K02121.1 and FJ445111.1), with multiple indels and SNPs. Because of commercial protections, we were unable to obtain the primer sequences used in the PCR kit to test this hypothesis further. This is also supported by the CT scores of the qPCR; all but one of the samples (054) had a CT of >31.0. The major conclusion is that there is inconsistency among PCR and confirmatory sequencing protocols, which will need to be addressed in the use of these methods for biosurveillance or diagnostics.

RSV (sample 052) and PIV (sample 131) were detected in one sample each by shotgun RNA sequencing, but not by PCR. There were 199 reads mapped to RSV by PanGIA, and only 11 reads mapped to PIV by PanGIA. It is unclear why PCR did not detect RSV in this sample, given the relatively high number of reads mapping to the reference genome in this case. When we mapped these reads to multiple RSV reference genomes, the best reference (GenBank entry: KJ723484.2) had a genome coverage of 86.5% with 86 SNPs. Similarly, for sample 131, we identified 4 regions that mapped to a PIV reference strain (GenBank entry: KY973568.1) and among these regions, there were 6 SNPs. We hypothesize that the negative results for PCR may be caused by the primers having enough mismatches to not bind sufficiently well for a positive call, again reiterating the need for robust primers and probes for PCR assays. We determined that these two samples were indeed true positives and were further confirmed with the more specific GOTTCHA2 taxonomic tool which also detected these viruses (RSV: 186 reads; PIV: 13 reads).

Once we had captured the alignment of outcomes between PCR and sequencing, we proceeded to compare the outcomes of these two laboratory methods with syndromic diagnostics. POC diagnostics exist only for a few of the commonly occurring respiratory pathogens, and are varied in sensitivity, specificity, and reliability. Out of all the individuals sampled, there were only 20 POC tests performed (10 influenza POC tests, and 10 tests for streptococcal infections). Five of the 10 POC flu tests were performed on individuals whose samples were also assessed by PCR and sequencing. Three of these five tests were negative by all three methods. One sample, (sample 120) was positive for influenza A by a POC test, PCR, and RNA sequencing (Table 3), again demonstrating concurrence between the three methods. A second individual (patient 140) was treated with antivirals after a positive POC flu test. However, this outcome was not supported by either PCR or RNA sequencing, and no respiratory viruses were identified using these methods (Table 3). This may be example of the poor specificity and/or sensitivity of the POC flu tests, although a greater sample number is required to illustrate this point effectively. Given that each patient was sampled only once, issues regarding sample collection and processing cannot be discounted.

As shown in Fig 4, 12/97 samples were true positives when sequencing was used as the true-reference test for evaluation of outcomes. Of these 12, only 1 (8.3%) was matched the physician’s diagnosis (patient 120; Table 3). Four individuals presented with symptoms of respiratory infection, but were not diagnosed with one by syndromic diagnosis. However, respiratory pathogens were identified by both sequencing and PCR (patients 080, 154, 128, 138; Table 3). These are clear examples of false negative syndromic diagnoses. Three individuals (patients 055, 129, and 150) were diagnosed with influenza by syndromic diagnostics; however, PCR and RNA sequencing did not detect influenza in these samples (Table 3).

Overall, 91.7% (11/12) false negativity rate is evidenced if relying only on syndromic diagnostics, which could result in significant consequences in the event of an unanticipated outbreak or a pandemic, as with COVID-19. The potential for misdiagnosis is readily understood when the commonality of symptoms and disease presentation among common respiratory pathogens is examined (Table 4). The symptoms of the individuals that tested positive by RNA shotgun sequencing for the five most common viruses are shown in Table 4. Percentages of individuals displaying symptoms were calculated for influenza A (n = 3 individuals), influenza B (n = 4), RSV (n = 8), coronavirus (n = 7), and PIV (n = 4). The significant overlap in syndromic presentation of respiratory pathogens will impact clinical diagnosis. Only one patient with influenza A presented with a fever, while none of the four patients with influenza B presented with a fever.

**Table 4:** Symptoms the individuals were experiencing at the time of the clinic visit for the 5 most common viruses detected in this study using RNA sequencing.

Virus	Cough	Sore throat	Fever	Runny nose	Ear ache	Body aches	Weakness, Fatigue	Headache	Congestion	Wheezing
Influenza A (n = 3)	100%	100%	33%	66%	33%	66%	100%	33%	100%	33%
Influenza B (n = 4)	75%	50%	0%	25%	25%	0%	25%	50%	25%	0%
RSV (n = 8)	100%	75%	0%	87.5%	25%	12.5%	37.5%	37.5%	87.5%	0%
Coronavirus (n = 7)	71.4%	71.4%	28.6%	85.7%	14.3%	57.1%	57.1%	57.1%	71.4%	14.3%
Parainfluenza (n = 4)	100%	75%	0%	75%	50%	25%	50%	75%	100%	0%

## Discussion

The current COVID-19 outbreak is testament to the emergence of novel respiratory pathogens that can cause outbreaks of pandemic potential around the world. The most used strategy for identifying infectious diseases at clinics around the world (including the U.S.) is syndromic diagnosis, which does not allow for discrimination between pathogens responsible for respiratory infections because of overlapping symptoms. Diagnosis of infection can greatly impact tailored treatment, community spread, and outbreak control. Yet, determining the efficacy of syndromic diagnosis as a method to identify and treat upper respiratory tract infections has not been given serious attention. The goal of this study was to overcome this gap and understand the bias in current syndromic diagnostic approaches during a typical annual respiratory infection season in the United States.

The first outcome of our study is methodological. We identified the presence of various viral pathogens in nasal and oral swabs, and that combining the two samples offers better outcomes for diagnosis of respiratory infection. However, a more controlled study would be needed to confirm this. Using nasal and oral samples for viral diagnostics may reduce the cost and time of extracting RNA, and subsequent signature measurement by sequencing or PCR type methods. Further, these samples are much more easily collected than nasopharyngeal swabs, which are more invasive, painful, and uncomfortable. Further, in certain viral diseases, viral shedding rate varies among individuals of different age groups and co-morbidities (immunocompromised, diabetics and others) [28]. By pooling nose and throat samples for each patient, the probability of obtaining RNA in low viral shedding individuals can potentially be increased. This would improve the sensitivity of detection by PCR, but not for sequencing, unless more reads are being sequenced per sample. This finding is supported by other studies in the literature, which suggest that combining nose and throat swabs are just as sensitive as nasopharyngeal swabs [29–32]. However, it is possible that certain pathogenic viruses were missed via selective sampling in only the nose and throat. Also, other investigators have shown that multiple sequencing libraries can generate variable results, and therefore, repeat measurements of such studies are also required to assess such effects.

The second major finding of the study was that only 36 (37.1%) of the individuals that presented with symptoms of respiratory infection (n = 97) were given a positive diagnosis for a respiratory infection or viral syndrome at the clinic. Point-of-care (POC) testing was not widely used to diagnose illness at the clinic. Indeed, of the 12 true positive individuals (i.e., individuals where PCR and RNA sequencing outcomes concurred for one or more of the 7 target viruses), only one sample had a POC test performed. The result of the POC testing in this single patient concurred with the doctor’s diagnosis as well as with the molecular results. Four individuals were not given a diagnosis by the physician, and 7 were given a diagnosis of some kind of upper respiratory syndrome. One patient that was diagnosed with the flu but was not subject to the POC flu test. Thus, there is significant misdiagnosis of viral respiratory pathogens at the point of care. The lack of reliance on POC testing is likely because of the unavailability or cost of such tests for routine use at the physician’s office. Methods such as PCR require the sample to be sent to a reference laboratory for diagnostic evaluation, often causing a delay of hours to days in obtaining the results, which is not conducive to the management and treatment of acute infectious diseases.

Third, we identified the most common viruses causing respiratory infection in northern New Mexico to be influenza (A and B), coronavirus, and RSV. Estimates show that rhinoviruses and enteroviruses alone account for half of upper respiratory tract infections [33, 34], which we did not observe here. Using shotgun RNA sequencing and taxonomic tools implemented within EDGE, we were able to match reads to three different strains of coronavirus. These strains are coronaviruses HKU1, 229E, and NL63. These three strains, along with a fourth (OC43) not detected here, are globally distributed and cause mild and more severe upper respiratory infections [35–39]. They can also be associated with more serious infections, such as pneumonia [40]. Moreover, studies have found that people can asymptomatically shed respiratory viruses [41]. Coronaviruses and adult RSV did not feature in the common diagnostic targets considered by the physician or POC diagnostics. As is evident with the COVID-19 pandemic, these pathogens have the ability to cause widespread and debilitating disease, and hence should be incorporated in the routine diagnostic evaluation at POC centers.

Fourth, we obtained an excellent corroboration between PCR and shotgun RNA sequencing for known viral pathogens, and showed better agreement than a previous study comparing PCR and next-generation sequencing [42]. Coronavirus, rhinovirus, and RSV were viruses that showed the most differences between the two methods. For instance, PCR detected rhinovirus in two individuals, which were not detected using RNA sequencing (false negative by sequencing). After obtaining reference genomes of rhinovirus, reads mapping to rhinovirus were determined to be of poor quality. Similar to the results regarding rhinovirus, we found that when sample reads were mapped to reference genomes, the mapped regions turned out to be of low complexity (coronavirus), or no reads mapped at all (RSV). Thus, false negative results may be likely using sequencing and should be assessed more systematically to avoid misdiagnosis. One solution to minimize false negative outcomes associated with sequencing is to sequence higher numbers of reads per sample (e.g., 40 million compared to 24 million reads per sample in this study). Viruses with few reads may not be sequenced given the amount of background, non-target sequences in the sample. However, sequencing this many reads on some of the lower output sequencers (e.g., MiSeq) would not be practical because only a limited number of samples could be run on one flow cell. Only two samples showed a positive result using shotgun RNA sequencing, but negative using PCR. Both samples contained relatively small numbers of reads matching respiratory pathogens (11 for PIV and 199 for RSV). Due to the nature of the reads and their alignments, these are interpreted as being true positives with false negative PCR results.

Our study was intended to be an exploration of the methods and strategies required for community surveillance, considerations for diagnostics development and deployment, and other factors. For instance, with the current COVID-19 pandemic, a lot of effort was spent on the choice of the sample, storage conditions, refining of methods, and data integration modalities in the early stages of the pandemic. Having a better assessment of surveillance strategies can help minimize such transition times, and better control spread within the surveillance window. Our sample size was sufficient to provide a window on the development of methods. Further, in terms of a shotgun sequencing study, we believe that the sample size was significant for methods evaluation. In addition, we had pooled nasal and throat samples from 156 individuals. Out of these, 97 were randomly selected for testing by commercial PCR. These sample sizes are in line with other studies comparing diagnostic tests and tools, including PCR and shotgun sequencing [43].

With increases in emerging and re-emerging of pathogens, antimicrobial resistance, and globalization and migration, the need for rapid and universal strategies for the diagnosis of infection has become critical [44]. The current COVID-19 pandemic, caused by a coronavirus, presents with the unique trifecta of asymptomatic carriers, aerosol transmission, and high infectivity, making itself ideally suited for pandemic spread [5, 45]. Coronaviruses were found to commonly circulate in the population tested in our study, and the mutagenicity and adaptability of these pathogens makes them emerging candidates for causing global pandemics. It is therefore important to acquire the tools to be able to accurately identify, characterize, and treat infectious diseases. This requirement extends beyond RNA viruses discussed in this paper to other viruses and bacterial pathogens [46].

Molecular detection assays, specifically PCR, are rapid and have replaced many traditional detection methods in clinical laboratories [47]. PCR-based methods have decreased the time to result for diagnostics to a few hours. However, a well-equipped reference or regional laboratory, trained technical personnel, and cold-chain processing is still required. In most clinical practices, shipping or sending the samples for PCR confirmation adds at least one day to diagnostic outcome confirmation, a factor which impedes their routine use by clinicians. This delay may account in part for the poor reliance on diagnostic confirmation noted in the present study. Highly sensitive and multiplexed syndromic PCR panels exist for respiratory pathogens, but the method still can only be applied to anticipated known agents and does not address emerging threats [48, 49]. In addition, updating and validating PCR panels may be required because of new strains and incompatible primers [50], especially with rapidly evolving influenza viruses [51]. The re-design of PCR-based assays for COVID-19 and the challenges therein, especially with the continued mutagenesis of the pathogen, is evident during the current pandemic [52].

Next-generation sequencing (NGS) has recently been investigated as an unbiased approach to detect known viruses and to discover novel viruses causing infections [42, 43, 53] and is being integrated into clinical laboratories for the purpose of aiding in infection diagnosis [54]. The use of this strategy clearly overcomes one of the key limitations of PCR, in that it can target both known and unknown pathogens. However, although the cost of NGS is decreasing, it is still relatively high compared to PCR-based detection assays, which must be considered when trying to use NGS in clinical settings. There is also a need for standardization of sample processing methods, and development of methods of detection and thresholds/parameters to identify pathogens present in a sample. Furthermore, sample preparation, library preparation, and bioinformatic analyses are time consuming, expensive and technically intensive, and account for some of the challenges associated with implementing NGS as a routine detection platform in clinical settings [54, 55]. The need to have a more standard and reliable approach to diagnose both common infections as well as documenting evidence of new pathogens that may lead to larger, more severe outbreaks is critical. A layered diagnostic strategy commencing with the availability of rapid POC technologies can greatly enhance our preparedness to future outbreaks. We suggest that using a combination of RNA sequencing and PCR be used as the ’true’ test, which would provide information regarding false negatives of both methods.

Our study finds that misdiagnosis associated with accurate identification of common respiratory pathogens in a clinical setting will continue to be high in the absence of POC diagnostics. An effective POC diagnostic strategy would be agnostic–broadly applicable to a range of known and unknown pathogens with equal efficacy. It should also be rapid, and easy to use at the point of need, so that timely results and decision making is feasible. Indeed, the outcomes of the diagnostic should ideally be available during the time of the consult with the physician, so that the therapeutic intervention is tailored accordingly. Further research into the development of such agnostic platforms, universal sample processing methods, bioinformatic pipelines, engineering solutions, and clinical evaluations is required to enhance our preparedness for the next pandemic. In the future, we hope to use the optimized approach for community surveillance developed here in larger cohorts, with the goal of exploring outcomes in the population.

## Supporting information

S1 Data Data for the 156 samples used in this study. For each sample listed, we provide data on patient information, symptoms at time of sampling, doctor’s diagnosis, and positive and negative PCR and RNA sequencing results. We also listed those samples that were used in the nose and throat sample comparison. The corresponding sample pair for the nose/throat comparison are listed below the first 156 samples.
(XLSX)
